# Patient experiences and perspectives of DMARD monitoring in Australians with long-disease-duration rheumatoid arthritis and psoriatic arthritis

**DOI:** 10.1186/s41927-025-00573-0

**Published:** 2025-10-23

**Authors:** Morgan Witts, Rachelle Buchbinder, Susan Lester, Jessica Stanhope, Vibhasha Chand, Claire Barrett, Rachel J. Black, Marissa Lassere, Lyn March, Paul Kubler, Catherine L. Hill, Philip C. Robinson

**Affiliations:** 1 Department of Medicine, Ipswich Hospital, Ipswich, QLD Australia; 2 Department of Medicine, The University of Queensland, Brisbane, QLD Australia; 3 Musculoskeletal Health and Wiser Health Care Units, School of Public Health and Preventive Medicine, Monash University, Melbourne, VIC Australia; 4 Rheumatology Unit, The Queen Elizabeth Hospital, Central Adelaide Local Health Network, Woodville South, South Australia Australia; 5 Adelaide Medical School, Faculty of Health and Medical Sciences, University of Adelaide, Adelaide, South Australia Australia; 6 School of Public Health, Faculty of Health and Medical Sciences, The University of Adelaide, Adelaide, South Australia Australia; 7 Department of Rheumatology, Redcliffe Hospital, Redcliffe, QLD Australia; 8 Rheumatology Unit, Royal Adelaide Hospital, Central Adelaide Local Health Network, Woodville South, South Australia Australia; 9 Department of Medicine, University of New South Wales, Sydney, NSW Australia; 10 Department of Rheumatology, St George Hospital, Kogarah, NSW Australia; 11 Florence and Cope Professorial Department of Rheumatology, Royal North Shore Hospital, St Leonards, NSW Australia; 12 Department of Rheumatology, Institute of Bone and Joint Research at Kolling Institute, University of Sydney, Sydney, NSW Australia; 13 Department of Rheumatology, Royal Brisbane & Women’s Hospital, Herston, QLD Australia

**Keywords:** DMARDs, Inflammatory arthritis, Psoriatic arthritis, Rheumatoid arthritis, Monitoring, Patient perspectives

## Abstract

**Background:**

Regular laboratory testing is recommended for monitoring disease activity and adverse effects in people taking conventional synthetic disease-modifying antirheumatic drugs (csDMARDs), yet the perspectives and experiences of patients have not been examined and may inform practice. As such, we aimed to determine the experiences and perceptions of laboratory monitoring in Australian adults using csDMARDs for rheumatoid arthritis (RA) or psoriatic arthritis (PsA).

**Methods:**

Participants in the Australian Rheumatology Association Database who had RA or PsA were sent an online questionnaire capturing their demographics, frequency of advised and actual blood tests, barriers, benefits and whether they felt they had been given an adequate rationale for testing or felt they needed more information. Data were reported using descriptive statistics.

**Results:**

The survey was distributed to 904 ARAD participants with RA and/or PsA with 679 responses (75.1%). A total of 451 people (348 RA, 103 PsA) fulfilled inclusion criteria. Most respondents were female (72.3%) with a mean age of 63.2 ± 10.7 years. The mean disease duration was 25.0 ± 11.7 years, and most respondents (82.5%) were taking methotrexate. Respondents reported being asked to have blood tests most frequently every 12 weeks (30.1%), followed by 4 (14.6%), 26 weeks (14.4%) and 8 (13.0%) weeks. 70.5% reported that they had their blood tests either early or on time. Most respondents (87.4%) reported that they felt the rationale for blood tests had been properly explained to them, with 19.5% reporting they would like more information. Overall, few reported perceived risks and costs, while almost all reported perceived benefits.

**Conclusion:**

Australian adults with RA and PsA taking csDMARDs are typically adherent to the schedule of laboratory monitoring requested by their treating rheumatologists. For most respondents, they reported perceived benefits with few reporting risks or costs. These self-reported findings from a long-disease-duration cohort should be used to inform standardised national guidelines for laboratory monitoring of csDMARDs. Further research into the perceived gaps in patient understanding of the need for laboratory testing may further enhance adherence.

**Supplementary Information:**

The online version contains supplementary material available at 10.1186/s41927-025-00573-0.

## Introduction

While a large body of evidence demonstrates the use of disease-modifying antirheumatic drugs (DMARDs) in inflammatory arthritis such as rheumatoid arthritis (RA) and psoriatic arthritis (PsA) is typically safe [1], harms can still occur. For example, conventional synthetic (cs)DMARDs are associated with myelosuppression and hepatotoxicity [2], with increased risks for those with co-morbidities, including existing renal impairment and fatty liver disease [3]. As such, laboratory monitoring is recommended to monitor treatment effect, and for early detection of adverse effects.

Keywords = DMARDs, inflammatory arthritis, psoriatic arthritis, rheumatoid arthritis, monitoring, patient perspectives

International guidelines regarding laboratory monitoring for people using csDMARDs are varied [4], and are largely based on expert consensus rather than research-based evidence. Recent developments suggest laboratory monitoring for 1–3 months after csDMARD initiation followed by “frequent” monitoring, of which frequent is not explicitly labelled [5]. In general, international recommendations suggest patients on csDMARDs have laboratory testing of these parameters in a step-down manner where testing commences every 2–4 weeks for the first 3 months after initiation of the csDMARD, then 8–12 weeks for 3–6 months, and every 12 weeks thereafter [4]. For some patients this testing regime may be challenging due to the cost and feasibility of having regular blood tests.

A recent study of rheumatologists and rheumatology trainees internationally found that 40% of participants felt that monitoring tests associated with the use of csDMARDs were conducted too frequently [6]. The participants reported similar types of tests, but a range of results regarding the frequency of laboratory testing. In general, people taking both methotrexate and leflunomide, and those with multiple comorbidities, are typically being tested more frequently than others [6]. A similar study found that healthcare professionals and their patients on disease-modifying therapy for inflammatory arthropathies agreed that current testing frequencies were burdensome [7]. Individualised risk-stratified monitoring plans were deemed acceptable, with monitoring frequency increased to biannually or annually depending on patients’ risk profiles [7]. 

In Australia, Medicare supports consumers by subsidising healthcare costs via the Medicare Benefits Schedule (which subsidises medical services) and Pharmaceutical Benefits Scheme (which subsides prescription medicines) [8]. Most routine laboratory tests incur no cost to patients, as provider commonly use “bulk-billing” whereby the provider is reimbursed directly by Medicare and the patient pays no out of pocket cost [9]. 

Patient perspectives are critical in understanding issues around adherence with prescriptions and referrals, including for laboratory monitoring. While previous research has examined consumers’ perspectives regarding csDMARDs themselves [10–13], perspectives and experiences of the associated laboratory monitoring have not been investigated.

The objective of this study was to determine the experiences and perceptions of csDMARD monitoring in Australian adults with RA and PsA who take csDMARDs. Specifically, we examined the frequency of blood tests (recommendations and practice), patient perceptions regarding the information provided about their blood tests, the costs of blood tests (both financial and time), and the self-reported perceived benefits and harms of blood testing to monitor their disease and/or adverse events associated with their use of csDMARDs. The findings of this study will assist in informing the development of drug monitoring recommendations within the NHMRC-approved Australian living guidelines for inflammatory arthritis [14, 15]. 

## Methods

A survey was developed by the authors and distributed to eligible participants of the Australian Rheumatology Association Database (ARAD). ARAD is a voluntary Australian registry that collates health information from patients with inflammatory arthritis that have been referred from their rheumatologist or self-referred for participation [16, 17]. ARAD was initially established in 2002 to determine the effectiveness and safety of biologics [16, 17]. Currently, ARAD participants are asked to complete questionnaires when they join, every 6 months for 2 years, and every 12 months thereafter. Participants in ARAD may be asked to complete additional, targeted questionnaires, as was the case for this study.

ARAD participants who met the following criteria were invited to participate in this study: age 18 years or older, current rheumatologist-diagnosed RA or PsA, have completed an ARAD survey online and consented to be contacted regarding participation in additional surveys. Participants were sent the survey irrespective of their medication status as it could not be assumed that current medications listed on ARAD were accurate at the time of reporting.

A link to the survey was sent to eligible ARAD participants in January 2023 with a reminder email sent 2 weeks later. The survey was closed after a total of 4-weeks. The survey collected information on current medications and the experience of adverse events, blood testing intervals to monitor medications and/or disease state, the provision of information related to blood tests, costs (financial and opportunities lost), and the perceived risks and benefits (Supplementary File 1). Data were collected using REDCap (Research Electronic Data Capture [18, 19]).

To supplement the survey data, the following data was extracted from the most recent routine ARAD survey: Self-reported demographics, current medications, education levels, and socioeconomic status (SES). Socioeconomic status was determined by participant’s address, classified into Statistical Area 1 (SA1). The Australian Bureau of Statistics assigns various socioeconomic scores to each postcode, based on Census data. In this study the Index of Relative Socioeconomic Advantage and Disadvantage (IRSAD) scores [20], was used for each SA1. The IRSAD scores were classified into quintiles from 1 (lowest) to 5 (highest) for each SA1 area [20], and these quintiles were then allocated to the participants. Data were also extracted from ARAD for the Single Item Literacy Screener (SILS), an instrument that asks participants how often they require assistance with reading health-related material, with a scores ranging from 1 (always) to 5 (never), was also extracted [21]. Data for the Assessment of Quality of Life Utility Index (AQoL [22]) were extracted. The AQoL assesses participants’ quality of life and ranges from 1.00 (full health) to 0.00 (death equivalent) [22]. Finally, data from the Beliefs about Medication Questionnaire (BMQ) were also extracted. The BMQ assesses response to general medication (overuse and harm) and treatment-specific (necessity and concerns) questions ranked from 1 (least favourable) to 5 (most favourable) [23]. These data were used to characterise the sample.

Data were analysed in Stata 18 [24] and reported using descriptive statistics.

### Ethics

This study had ethics approved by Metro North HREC A (EC00172) (Reference: LNR/2021/QRBW/73446). Informed consent was obtained from all participants. ARAD has ethics approval from Monash University and the Central Adelaide Local Health Network.

## Results

The survey was sent to 904 ARAD participants with RA and/or PsA. There were 679 responses, giving a response rate of 75.1%. Of these, 451 (346 RA, 103 PsA) were currently taking csDMARDs. Most respondents were female (326/451, 72.3%) and the mean age was 63.2 ± 10.7. Table 1 describes the demographic and clinical characteristics of the 451 eligible respondents.

In comparison with survey non-respondents, survey respondents were similar in terms of diagnosis, gender, age at diagnosis, age at time of survey, disease duration, SES and geographical location. However, compared to non-responders, survey respondents were more likely to have had biologic or targeted synthetic DMARDs (90% v 84%) and received further education beyond secondary education (63% v 54%; see Supplementary File 2).

**Table 1:** Demographic and clinical characteristics of respondents (*n* = 451)

Characteristic	Mean ± SD
Age (years)	63.2 ± 10.7
*Disease duration (years)	25.0 ± 11.7
*Age at diagnosis (years)	39.1 ± 13.4
	**n (%)**
Type of arthritis	
* Rheumatoid*	348 (77.2)
* Psoriatic*	103 (22.8)
Gender	
* Females*	326 (72.3)
* Male*	124 (27.5)
* Non-binary*	1 (0.2)
*Treating rheumatologist’s sex (%)	
* Male*	224 (49.7)
* Female*	222 (49.2)
* Don’t know*	1 (0.2)
* Don’t see one*	4 (0.9)
Self-reported distance travelled to visit rheumatologist	
* 0–10 km*	158 (35.0)
* 10–50 km*	174 (38.6)
* 50–100 km*	40 (8.9)
* > 100 km*	72 (16.0)
* Not sure*	7 (1.6)
Current csDMARDs used	
* Azathioprine*	7 (1.6)
* Hydroxychloroquine*	96 (21.3)
* Leflunomide*	51 (11.3)
* Methotrexate*	372 (82.5)
* Mycophenolate*	1 (0.4)
* Sulfasalazine*	56 (12.4)
*Education level	
* Didn’t complete high school*	53 (11.8)
* Completed high school*	97 (21.5)
* Tertiary (University*,* TAFE*,* CAE)*	301 (66.7)
*Socioeconomic status (IRSAD quintile)	
* 1 (Lowest)*	86 (19.2)
* 2*	99 (22.2)
* 3*	89 (19.9)
* 4*	84 (18.8)
* 5 (Highest)*	89 (19.9)
*Frequency of some reading difficulties with health-related materials (SILS)	
* Never*	266 (82.1)
* Rarely*	41 (12.7)
* Sometimes*	15 (4.6)
* Often*	1 (0.3)
* Always*	1 (0.3)
	**Median (IQR)**
*Quality of life (AQoL-Utility Index)	0.62 (0.45–0.78)
*Beliefs regarding medications	
* Necessity of DMARDS*	4.2 (3.6–4.8)
* Concerns regarding DMARDS*	2.4 (2.0-2.8)
* Overuse of medications in general*	2.3 (2.0–3.0)
* Harms of medications in general*	2.2 (1.8–2.6)

*indicates data were obtained from the Australian Rheumatology Association Database (ARAD). SD: standard deviation; DMARD: disease-modifying antirheumatic drug; csDMARD: conventional synthetic disease-modifying antirheumatic drug, bDMARD: biologic disease-modifying antirheumatic drug; TAFE: Technical and Further Education; CAE: College of Advanced Education; IRSAD: index of relative socioeconomic advantage and disadvantage; SILS: Single Item Literacy Screener; AQoL: Assessment of Quality of Life; IQR: interquartile range

Nearly all respondents (98.7%, 445/451) self-reported being asked to have regular blood tests. The five respondents who reported they were not asked to have regular blood tests were taking hydroxychloroquine (*n* = 2) and methotrexate (*n* = 3). One additional participant indicated they were unsure whether they were asked to have regular blood tests; they were taking hydroxychloroquine and sulfasalazine. The frequency of advised blood testing reported ranged from every 1 to 52 weeks, with the most common frequencies being 12 weeks (134/445, 30.1%), followed by 4 weeks (65/445, 14.6%), 26 weeks (64/445, 14.4%) and 8 weeks (58/445, 13.0%).

**Fig. 1:**
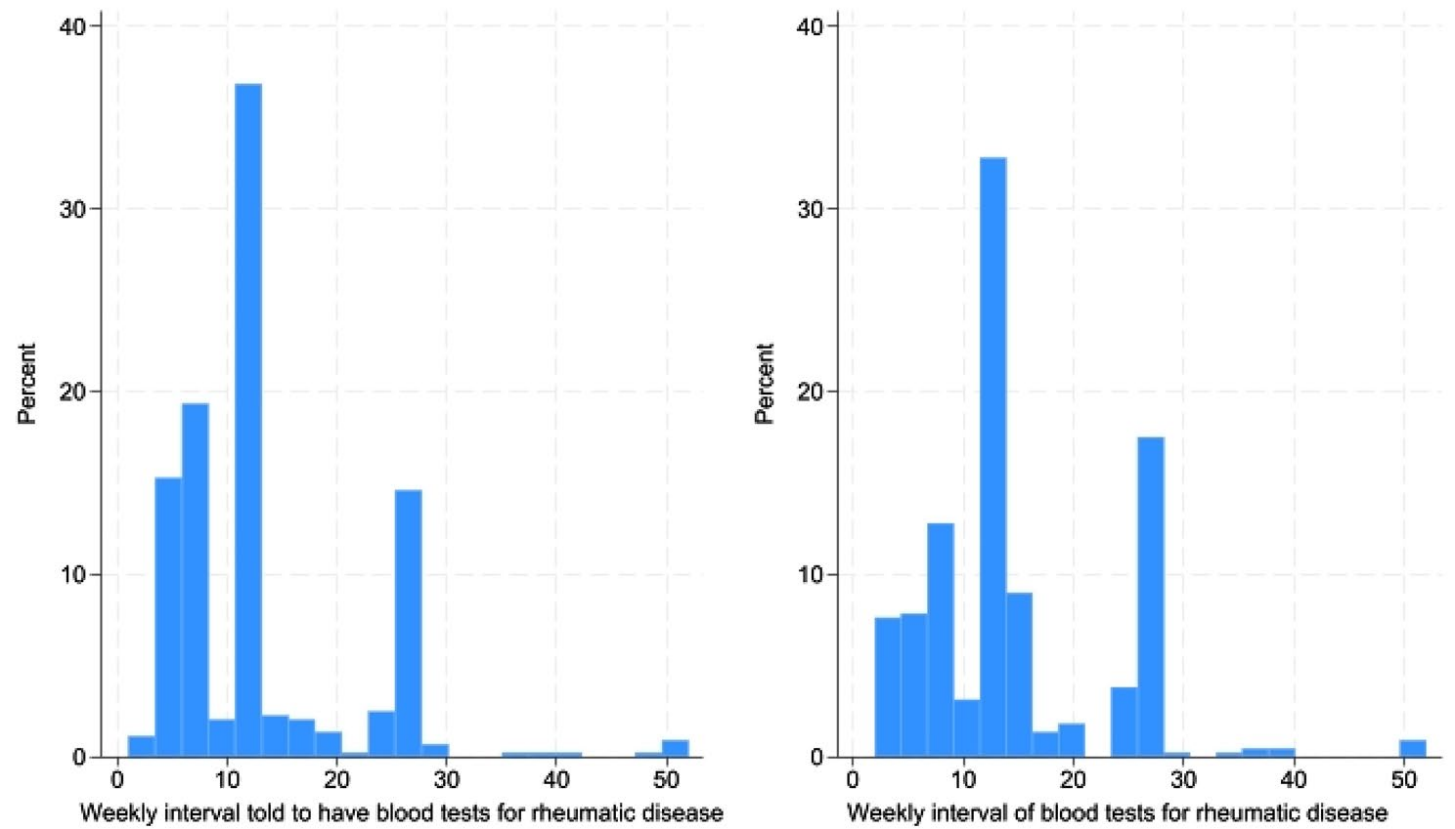
Distribution of weekly intervals comparing time asked to have a blood test and time physically having blood test (*n* = 451)

Most respondents (298/445, 67.0%) self-reported complying with the frequency of advised blood tests as advised by their treating rheumatologist. 16 respondents (3.6%) reported having blood tests more frequently than advised. Among respondents who did not have their blood tests as frequently as advised, delays in blood tests ranged from 1 to 23 weeks late, with the most reported late periods being 4 weeks (27/445, 6.1%), 2 weeks (24/445, 5.4%) and 1 week (23/445, 5.2%) (Fig. 1).

Most respondents (394, 87.4%) self-reported that they felt the rationale (e.g. the benefits and risks) of regular monitoring blood tests was properly explained to them, with 19.5% (88/451) indicating that they would like more information explaining why they are required to have regular blood tests.

Only eight respondents (1.8%) reported paying an out-of-pocket fee for their blood tests ranging from A$3 to $80, with the median being A$50.50 (IQR A$27.50–75.00). Another seven (1.6%) were unsure.

Table 2 highlights key responses to survey questions focused on travel requirements, opportunity loss and perceived risks and benefits. Most participants (54.6%) were within 30 min of a pathology centre, while the vast majority reported no opportunity loss or perceived risks related to monitoring (83.4% and 90.2%, respectively). The most reported perceived benefits were for monitoring hepatotoxicity (91.7%) and disease activity (88.7%).

**Table 2:** Travel requirements, opportunity loss, and perceived risks and benefits related to monitoring

Topic	*n* (%)
Distance travelled to pathology centre	
* ≤30 min* * 30 min–1 h* * 1–2 h* * >2 h* * Unsure*	246 (54.6) 147 (32.6) 147 (11.8) 4 (0.9) 1 (0.2)
Opportunity loss	
* Work* * Hobbies* * Socialising* * Study* * None*	45 (10.2) 30 (6.7) 21 (4.7) 3 (0.7) 378 (83.4)
Perceived risks	
* Injury* * Incorrect results* * Environmental damage* * None*	26 (5.8) 12 (2.7) 9 (2) 407 (90.2)
Perceived benefits	
* Monitor disease activity* * Hepatotoxicity monitoring* * Myelosuppression monitoring* * None*	400 (88.7) 413 (91.7) 319 (70.7) 2 (0.4)

## Discussion

In Australia, this study examined adults’ experiences and perceptions of laboratory monitoring associated with using csDMARDs for RA and PsA. Overall, we found in an Australian context, a self-reported range of blood test frequencies suggested to patients, but overall good adherence, with 70.5% reporting having their blood tests early or on time. The costs, related to financial, time and opportunity loss were low. Most respondents indicated benefits of laboratory monitoring, with few harms indicated. Together these findings indicate consumer acceptance of laboratory monitoring associated with the use of csDMARDs.

Consistent with testing recommendations [4], almost all respondents reported having regular blood tests, with the two most common time periods being 4 and 12 weeks. These frequencies are consistent with international recommendations, but are less frequent than Australian recommendations for most csDMARDs [14, 15]. Variability between recommendations and guidelines is prevalent throughout literature and highlights the need for implementing standardised guidelines to inform practice for laboratory monitoring frequency [6]. As our sample had a mean disease duration of 25 years, less frequent follow-up is expected, assuming no recent changes in the use of csDMARDs. Such challenges to monitoring recommendations are supported by observed low frequency abnormalities in laboratory results in long-term DMARD consumers [25]. The relatively low frequency of testing may be due to stable disease and treatment, with the clinician determining that more frequent monitoring is not required. These findings complement those of a recent study, where patients and healthcare professionals created individualised monitoring plans which saw testing intervals increased to three-monthly, biannually or annually [7]. The observed increased monitoring, interval coupled with high patient adherence, suggest that future living guidelines would benefit from incorporating shared-decision making and risk-stratified monitoring plans in stable patients.

Encouragingly, adherence with rheumatologist recommendations with the frequency of testing was reportedly high. Most respondents indicated they felt they were provided with adequate information related to the need for testing, almost all reported benefits related to the monitoring of disease activity or adverse effects related to the hepatotoxicity and myelosuppression, and majority reporting no risks related to injury, incorrect results, confidentiality risks nor environmental damage. Furthermore, our respondents generally incurred no out-of-pocket costs, and more than half travelled less than 30 min to the blood collection facilities. It is also possible that rheumatologists assess the patient’s likely adherence to monitoring when making decisions about medications; hence those who are unlikely to be adherent with blood tests may be less likely to be prescribed certain csDMARDs. The extent to which non-adherence to laboratory monitoring for csDMARDs alters prescription habits is yet to be fully explored in literature.

Most respondents indicated that they felt the need for regular blood tests had been properly explained to them, while only 19.5% indicated that they would have liked more information. Future studies could examine what these gaps are to better inform rheumatologists practice in discussing csDMARDs and the recommended monitoring with their patients. It is possible that patients requiring more information represent a subset of patients with more complex medical and socioeconomic backgrounds and therefore requires further exploration. These findings support those of previous studies, demonstrating patients in general have high levels of confidence in information conveyed by their rheumatologist [6, 26, 27]. For example, a recent study of patients with PsA found they were generally satisfied with their clinicians’ communication [28], however, similar to our findings, some wanted more information, and felt that asking more questions may impact their care. Notably, our sample was restricted to people who were able to complete the survey online, hence our sample may be more equipped to source their own information online, particularly given the sample was dominated by those with a tertiary level education (66.7%), with 94.8% indicating that they never or rarely have reading difficulties with health-related materials.

Given the generally positive reports regarding the communication related to the need for laboratory monitoring, it is perhaps not surprising that most respondents felt the benefits of blood testing included monitoring of disease activity, or for adverse effects relating to hepatotoxicity and myelosuppression. Similarly, few (9.8%) identified potential harms related to blood tests, related to injury, confidentiality risks, incorrect results, or environmental damage. Future studies should explore a broader range of potential benefits and harms using qualitative approaches to identify additional factors from a consumer perspective, not captured in our survey.

The use of a national cohort which included patients from a variety of care settings and a high response rate add to the strengths of the study. However, as discussed above, our respondents had a relatively long disease duration (mean 25 years); hence these findings are not necessarily generalisable to those in the earlier stages of RA and PsA, nor those with other forms of inflammatory arthritis or on biologic or targeted synthetic DMARDs (b/tsDMARDs). We therefore recommend targeted studies of these groups be undertaken. As with all studies of this nature, recall bias may have influenced these results, particularly given respondents were first asked to indicate how frequently they were asked to have blood tests done, before being asked how often they were performed. As this study relies on self-report, it is possible that patients overstated how frequently they underwent monitoring as previous studies have shown that non-completion rates for blood tests can range from 10 to 30% [29, 30]. Furthermore, these findings may have been influenced by selection bias, where those volunteering to participant in ARAD and those who agreed to undertake this specific survey may be different from the broader patient population. We excluded participants who were solely on b/tsDMARDs as the focus of this study was on csDMARD monitoring. We acknowledge that consumers may not be aware of the distinction between medication types. Future studies could examine rheumatologist-advised and actual test frequency to overcome these potential limitations.

## Conclusion

Adults with long-disease-duration RA and PsA in Australia are typically adherent with laboratory monitoring frequency for disease activity and adverse effects associated with the use of csDMARDs. Yet, the reported advised frequency for testing was lower than Australian recommendations. These findings provide important insights into the perceptions and experiences of people taking csDMARDs for their RA and PsA and should be used to inform living guidelines for monitoring csDMARDs.

## Supplementary Information

Below is the link to the electronic supplementary material.

Supplementary Material 1

## Data Availability

Data is provided within the manuscript or supplementary information files.

## Abbreviations

AQoL: Assessment of Quality of Life Utility Index
ARAD: Australian Rheumatology Association Database
BMQ: Beliefs about Medication Questionnaire
b/tsDMARDs: Biologic or targeted synthetic disease-modifying antirheumatic drugs
csDMARDs: Conventional disease-modifying antirheumatic drugs
DMARDs: Disease-modifying antirheumatic drugs
IRSAD: Index of Relative Socioeconomic Advantage and Disadvantage
Metro North HREC A: Metro North Health Human Research Ethics Committee A
PsA: Psoriatic arthritis
RA: Rheumatoid arthritis
REDCap: Research Electronic Data Capture
SA1: Statistical Area 1
SES: Socioeconomic status
SILS: Single Item Literacy Screener
