# Supplementary material for: Patient experiences and perspectives of DMARD monitoring in Australians with long-disease-duration rheumatoid arthritis and psoriatic arthritis
Source: BMC Rheumatol. 2025 Oct 23;9:126. doi: 10.1186/s41927-025-00573-0 (PMC12548214; doi:10.1186/s41927-025-00573-0)
Supplement: Supplementary file 1 — Supplementary Material 1 [file 41927_2025_573_MOESM1_ESM.docx]

**Supplementary Material:**

**Supplementary Material 1: Survey.**


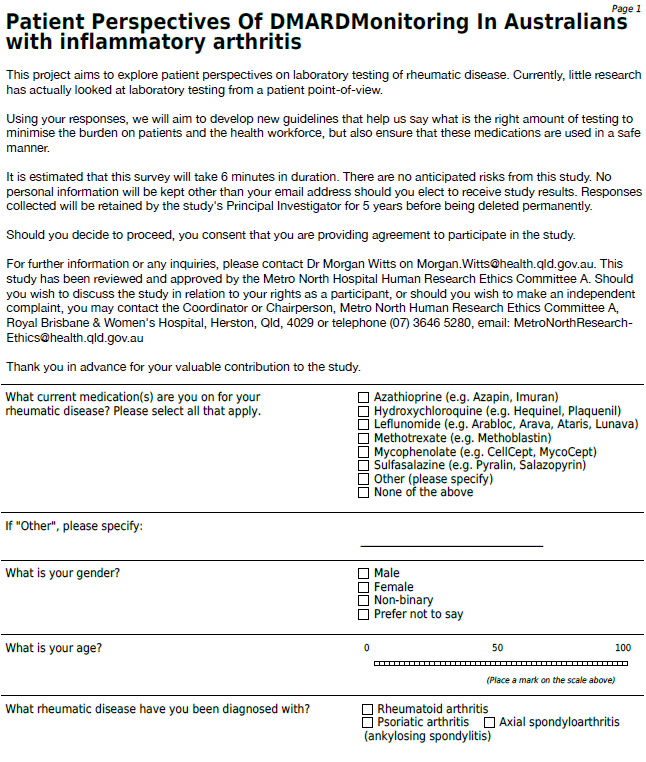


**Patient experiences and perspectives of DMARD monitoring in Australians with rheumatoid arthritis and psoriatic arthritis**


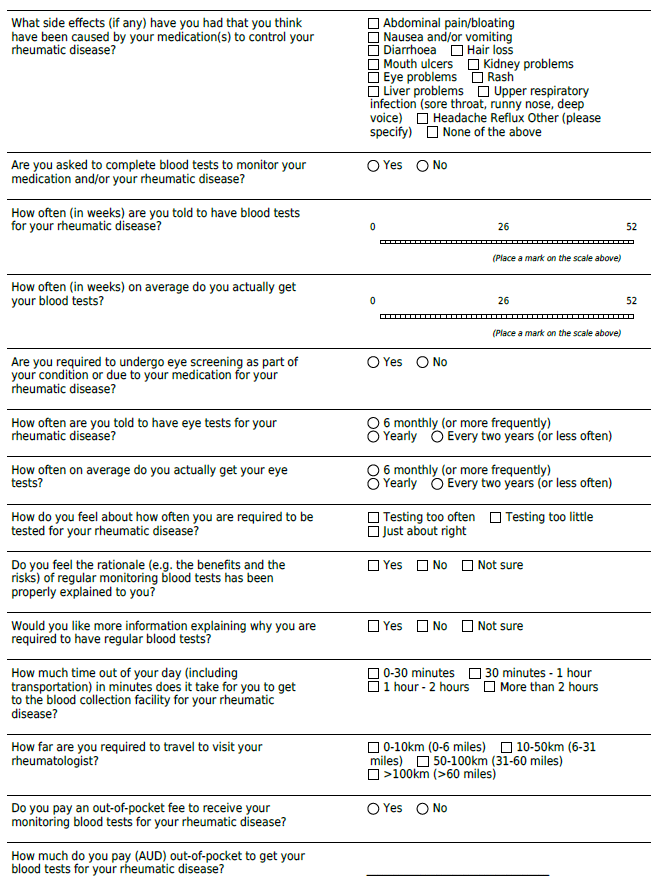


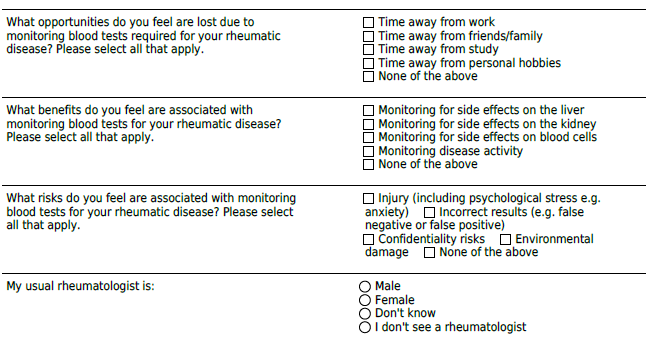


**Supplementary Material 2: Comparison of eligible non-responders versus responders.**

| Comparator | RA + PsA | |  | p-val |
| --- | --- | --- | --- | --- |
|  | Non-responder | Responder | ALL |  |
|  | n = 225 | n = 679 | N = 904 |  |
| Psoriatic Arthritis | 56 (25%) | 167 (25%) | 223 (25%) | 0.93 |
| Age at survey | 64 (12) | 64 (11) | 64 (11) | 0.74 |
| Disease duration | 22 (11) | 23 (12) | 23 (11) | 0.34 |
| ARAD duration | 11 (5) | 12 (5) | 12 (5) | 0.001 |
| Age at diagnosis | 42 (14) | 41 (14) | 41 (14) | 0.60 |
| Female | 163 (72%) | 496 (73%) | 659 (73%) | 0.86 |
| ARAD DMARD History |  |  |  |  |
| *csDMARDS_ever* | 202 (90%) | 628 (92%) | 830 (92%) | 0.20 |
| *b/tsDMARDS_ever* | 189 (84%) | 611 (90%) | 800 (89%) | 0.016 |
| *any DMARDS_ever* | 220 (97.8%) | 676 (99.6%) | 896 (99.1%) |  |
| Education (post HS) | 121 (54%) | 431 (63%) | 552 (61%) | 0.010 |
| Regional/Remote | 81 (36%) | 222 (33%) | 303 (34%) | 0.38 |
| IRSAD AUS_quintiles |  |  |  |  |
| *Q1* | 47 (21%) | 122 (18%) | 169 (19%) | 0.90 |
| *Q2* | 45 (20%) | 143 (21%) | 188 (21%) |  |
| *Q3* | 45 (20%) | 138 (20%) | 183 (20%) |  |
| *Q4* | 43 (19%) | 128 (19%) | 171 (19%) |  |
| *Q5* | 44 (20%) | 143 (21%) | 187 (21%) |  |
